# The Impact of Intervention on Sexual Practices of HIV Positive Individuals in Southeast Nigeria

**DOI:** 10.1155/2009/127480

**Published:** 2010-01-26

**Authors:** S. N. Obi, N. A. Ifebunandu, H. E. Onah, A. K. Onyebuchi

**Affiliations:** ^1^Department of Obstetrics and Gynaecology, University of Nigeria Teaching Hospital, Enugu 400001, Nigeria; ^2^Department of Medicine, Federal Medical Centre Abakaliki, Enugu 400001, Nigeria; ^3^Department of Obstetrics and Gynaecology, Federal Medical Centre Abakaliki, Enugu 400001, Nigeria

## Abstract

*Objective*. To describe the impact of repeating behavioral educational intervention on sexual practices of HIV positive individuals. *Method*. A prospective cohort study of HIV positive individual was conducted in southeast Nigeria from June 2007 to May 2008. Information on sexual practices was collected at initial visit; education was given and its impact was evaluated afterwards. *Results*. Knowledge about risk of unprotected intercourse increased by 41%, condom use by 27% (*P* < .001) and consistent condom use by 55% (*P* < .001). The significant predictors of consistent condom use include male gender, multiple sexual partner, as well as good knowledge of HIV transmission, higher educational status and being married. Non use of condom at postintervention survey were characterized by female gender (*n* = 4), monogamous relationship (*n* = 10), little or no education (*n* = 10), and unmarried (*n* = 7) respondents. *Conclusion*. Repeated behavioral education intervention improves consistent condom use among HIV positive individuals and will help curb the spread of HIV/AIDS.

## 1. Introduction

HIV/AIDS are major challenges to health and development the world over. This is worse in developing countries including Nigeria where several factors including polygamy, high prevalence of untreated sexually transmitted infections (STIs), low condom use, poverty, low literacy, poor health status and stigmatization contributed to the rapid spread of HIV/AIDS [1–6]. Currently, more than 80% of reported HIV sero-positive individuals in developing countries including Nigeria were infected through heterosexual transmission [7]. The previous finding [8] of high risk sexual practices among HIV positive individuals showed potentials risk for the rapid spread of HIV. 

 In response to the increased prevalence of HIV infection in Nigeria [9] and the knowledge that consistent condom use provides protection from HIV and STIs [10], we developed an intervention programme which included a prospective cohort study for HIV positive individuals attending HIV clinic. The programme, the first educational intervention of the kind to target Nigerian HIV positive individuals focused on knowledge about HIV transmission, safe sexual practices and increasing condom use in a bid to curb the spread of HIV in Nigeria.

## 2. Materials and Methods

This study carried out at Federal Medical Centre Abakaliki, Ebonyi state and University of Nigeria Teaching Hospital Enugu, Enugu state *was* an extension of previous study on sexual practices of HIV positive individuals in southeast Nigeria [8]. *The Federal Medical Centre at Abakaliki, a growing urban town and capital of Ebonyi state is one of the two tertiary health institutions that serve the whole state. The second health institution, University of Nigeria Teaching Hospital, located at Enugu, capital of Enugu state is a referral health centre for the whole of the southeast Nigeria. The patients from both institutions are believed to be representative for the population in the region*. From June 2007 to May 2008, an educational intervention with ongoing recruitment during the intervention period was conducted in adult HIV and Prevention of Mother-To-Child Transmission (PMTCT) of HIV/AIDS clinics in both health institutions focusing on HIV positive individuals. The nature of the interventional programme was explained to the patients and all consenting HIV positive individuals were interviewed using self administered questionnaire. *The seven (3.8*%*) illiterate patients in the study were interviewed by trained nurses using standardized questionnaire translated in local Igbo language and subsequently they completed the questionnaire with the patients' responses. As much as possible, translators are of the same sex and within the same age group as patients so as to reduce bias*. Information sort included socio-demographic characteristics, duration of HIV diagnosis, sexual practices and knowledge of HIV transmission, condom use and the preventive role of condom in HIV transmission. All patients with AIDS and pregnant HIV positive women were excluded from the study. 

 After administering the initial questionnaire, *which provided the baseline data*, group counseling was provided on safe sexual practices, HIV transmission and prevention and condom use by trained doctors and nurses. The counseling emphasized the nature of HIV virus and the route of transmission during sexual intercourse. Also factors like skin ulcerations; genital sores, and so forth. which increases the risk of transmission were highlighted. The need to reduce risk of contracting other strains of HIV and other sexually transmitted infections by having sex with only one partner and using condom every time or abstaining from sex were emphasized. Also some postcoital practices like washing the vagina after ejaculation of semen were highlighted as ineffective in preventing HIV transmission as well as the fact that a healthy HIV positive individual can still transmit the virus during unprotected sexual intercourse. The counseling is repeated for individuals during consultation with their doctors. At the followup visits the counseling was the same as at the intake visit. Each of the respondents had at least three such counseling and the questionnaires were re-administered on the respondents six months after the intake visit. Each of the patients was given an identification code and the questionnaires were marked only with this code to guarantee confidentiality. On completion, the questionnaires were left in the box provided. The data obtained from the survey were analyzed by means of simple percentages, descriptive and inferential statistics. Statistical software SPSS for window version 10 was used in the analysis. The study received ethical approval from the hospitals' Ethics committees. 

 The information on condom use *(measured “always” (100*%*), “frequently” (50*%–*99*%*), “rarely” (1*%–*49*%*) and “never” (0*%*)) was regrouped into consistent (100*%*) and inconsistent (1*%–*99*%*)*. The respondents that never used condom *(0*%*)* were grouped as practicing unprotected intercourse. Knowledge about HIV transmission and risk of unprotected sexual intercourse were measured by asking respondents whether they agreed or disagree with some statements (see Supplementary Material available at doi:10.1155/2010/127480). Those who scored correctly on more than 70% of the statements were classified as having good knowledge and others as having poor knowledge. *The 70*% *score was chosen as cut-off based on the average score of randomly selected health workers in the same institutions who were administered with the same questionnaire on Knowledge about HIV transmission.*

## 3. Results

The study enrolled 220 HIV positive individuals, of whom 22 did not complete the questionnaires. After excluding 14 questionnaires with incomplete data, there were 184 respondents in the pre-intervention survey consisting of 88 (47.8%) male and 96 (52.2%) female while the post-intervention survey consisted of 85 (49.4%) male and 87 (50.4%) female. A total of 12 patients dropped out of the study either due to death or relocation of their place of abode. The mean age of respondents was 33.4 (±8.2) with a range 20–58 years. Seventy (38%) of them were single, another seventy (38%) married and the remaining forty four (23.9%) either divorced or separated from their spouse. The patients had been HIV positive for 1–72 (mean 13.03 ± 12.84) months. One hundred and four (56.5%) of the respondents were sexually active at the time of enrollment into the study and they were still sexually active at the post-intervention survey. Vaginal sex was the commonest (96.5%) type of sex. 

 All the respondents knew about condom *as a barrier contraceptive* and the proportion with good knowledge about the risk of unprotected intercourse increased from 39.7% (*n* = 73) before the intervention to 80.8% (*n* = 139) after the intervention. Prior to the intervention, 40.4% (*n* = 42) respondents reported non use of condom with partner, while among the 60% using condom, one quarter (*n* = 15) are consistent with it and the other three quarter (*n* = 47) inconsistent. The intervention resulted in significant increase (*P* < .001) in the number of respondents using condom from 59.6% (*n* = 62) to 86.5% (*n* = 90) pre- and post- intervention respectively. Consistent use of condom also showed a three fold increase from 24.2% (*n* = 15) before the intervention to 78.9% (*n* = 71) after the intervention (*P* < .001). The mean number of sexual partners per respondents before and after the intervention showed a reduction from 0.8 ± 6 to 0.6 ± 5. The characteristics of the 14 respondents who still reported non-use of condom at the post-intervention survey are female (*n* = 10), those with single sexual partners (*n* = 10), little or no education (*n* = 9), unmarried (*n* = 7), and sero-concordant couple (*n* = 2). 

Table 1 showed the showed the predictors of consistent condom use among these HIV positive individuals. 

## 4. Discussion

The level of unprotected sexual intercourse among HIV positive individuals prior to the intervention was high. Giving the risk to which these HIV positive individuals expose themselves and their partners especially the sero-negative ones, an educational intervention on safe sexual practices and consistent condom use is timely. This intervention to reduce risk of HIV transmission resulted in greater condom use and increase in proportion of respondents with good knowledge of HIV transmission and prevention at the post intervention survey. 

 This study with repeating intervention, the first of its kind in Nigeria suggested that an educational programme on condom promotion may have a considerable impact on increasing consistent condom use. With stigmatization and discriminatory attitude towards people living with HIV/AIDS in Nigeria [4–6], it is difficult to approach these individuals *in the community setting* with preventive efforts and safe sexual practices. *Furthermore, the public campaign and advertisement on HIV and its prevention, often carried in the electronic media do not get to majority of the populace because of poor access to such facilities*. This could account for the poor knowledge of HIV found at baseline among our respondents. The impressive increase we found with consistent condom use during the follow up period demonstrated how efficacious such intervention programme for HIV positive individual in Nigeria may be. In this population of HIV positive individuals, good knowledge of HIV transmission and multiple sex partner were the strongest predictors of consistent condom use and this was in agreement with previous study from Nigeria [11]. With such good knowledge, respondents that engage in risky sexual practices like multiple sex partners will appreciate the need for consistent condom use to prevent HIV and STIs. Higher educational attainment which no doubt will increase the knowledge of HIV transmission is also a significant predictor of consistent condom use. That the use of condom in this study is dependent on the male is a common finding in most cultural setting in Sub-Saharan Africa as the women cannot negotiate for its use because of hindered communication between partners on issues of sex [12]. 

 The study has some limitations. A prospective *cohort* study with one group is not the optimal design for evaluating intervention *effectiveness because it is vulnerable to effect of testing and history and may overestimate intervention effects [13]. This is better measured with a randomized trial with a control group [14]*. Despite emphasis on the importance of providing honest information to the respondents, the reliance on self reported sexual practices and condom use is another weakness common to many behavior intervention studies. *This is because it is not known whether patients who participated in the study were more predisposed to changing their behavior than non-participants. Also the issue of bias cannot be excluded for the illiterate patients that had face-to- face interview with the translators as against placing answers anonymously on paper. Furthermore, reliability of the answers from patients' that used translators was solely dependent on the translators*. Finally, being a short intervention, the ability of the study to evaluate the longer effect of the intervention is limited. 

 In conclusion, the study showed that a repeated behavioral education intervention improves consistent condom use among HIV positive individuals and will help curb the spread of HIV/AIDS. *On a broader scale, there is need for more public health educational campaign targeted at HIV positive, negative and those of unknown status in the community on HIV awareness, need for voluntary counseling and testing, preventive practices and treatment options for those found to be HIV positive. The community and village heads will play a role in mobilising the grassroots for this campaign*. 

## Supplementary Material

Questions used in assessing socio*‐*demographic characteristics and sexual behaviour of respondents as well as their knowledge of HIV transmission.

## Figures and Tables

**Table 1:** Predictors of consistent condom use.

	Consistent (*n* = 71)		Inconsistent (*n* = 19)		*P*-value
	No.	(%)	No.	(%)	
Marital status:					
Single	10	(14.1)	8	(42.1)	.0048^(a)^
Married	42	(59.2)	4	(21.1)	
Divorced/Separated	19	(26.8)	7	(36.8)	

Age					
<30	29	(40.8)	9	(47.4)	.6129^(b)^
>30	42	(59.2)	10	(52.6)	

Educational status:					
None	1	(1.4)	4	(21.1)	.0022^(a)^
Primary	5	(7.0)	3	(15.8)	
Secondary	24	(33.8)	7	(36.8)	
Tertiary	41	(57.7)	5	(26.3)	

Good knowledge of HIV transmission:					
Yes	59	(83.1)	2	(10.5)	<.0001^(b)^
No	12	(16.9)	17	(89.5)	

Number of sexual partners:					
1	25	(35.2)	15	(78.9)	.0013^(b)^
≥2	46	(64.8)	4	(21.1)	

Sex:					
Male	45	(63.4)	6	(31.6)	.0186^(b)^
Female	26	(35.2)	13	(68.4)	

^(a)^Using the *x* ^2^ test.

^(b)^Using the Fisher exact test
