# Supplementary material for: The Impact of Intervention on Sexual Practices of HIV Positive Individuals in Southeast Nigeria
Source: Obstet Gynecol Int. 2010 Jan 26;2009:127480. doi: 10.1155/2009/127480 (PMC2817496; doi:10.1155/2009/127480)
Supplement: Supplementary file 1 — Questions used in assessing socio‐demographic characteristics and sexual behaviour of respondents as well as their knowledge of HIV transmission. [file 127480.f1.pdf]

APPENDIX A

1. Age ----------- years
2. Sex: Male□   Female□
3. Marital status: Single□   Married□   Divorced□   Separated□
4. Religion: Christianity□   Moslem□   Traditional religion□   Others-------
5. Highest educational attainment: None□   Primary□   Secondary□
   Tertiary/University□
6. Your occupation--------------------------
7. Duration of HIV diagnosis------------------ months.
8. Are you sexually active? Yes□   No□
9. Number of sex partners since diagnosis of HIV --------------------
10. Type of sex: Vaginal sex □   Oral sex□   Anal sex□   Others (specify)-----
11. Ever heard of condom?   Yes□   No□
12. Ever used condom since HIV diagnosis? Yes□   No□
13. If yes to Q.12, how would you rate your use of condom
    (a) Use it for all sexual activity (100%)
    (b) Use them frequently during sexual activity (50-99% of the time)
    (c) Use them occasionally during sexual activity (1-49% of the time)
14. Do you know the HIV status of your sexual partner? Yes□   No□
15. Do your sexual partners know your HIV status? Yes□   No □

Statements about HIV transmission (use Agree, Disagree or Not sure)

16. HIV is transmitted through:
    (a) Sharing sharp objects like needle, razor e.t.c
    (b) Blood contact or transfusion
    (c) Unprotected sex with infected partner
    (d) Infected mother to unborn child

17. Healthy looking HIV infected individual cannot transmit HIV during unprotected intercourse (i.e. having sex without condom)

18. Sexual intercourse without condom do not expose me to any risk since I am already infected

19. If a woman washes her vagina after HIV positive individual has ejaculated into it, she cannot get HIV/AIDS.
